# Assessing the Utility of Automated and Pen‐And‐Paper Cognitive Assessment Tools for Underrepresented Groups in the UK

**DOI:** 10.1002/gps.70224

**Published:** 2026-06-06

**Authors:** Caitlin H. Illingworth, Madhurananda Pahar, Dorota Braun, Lise Sproson, Hina Khan, Bahman Mirheidari, Ronan O'Malley, Sahra Abdi, Muse Jama, Ismail Yussuf, Candice Wang, Sai Lee, Sarah Ng, Nur Ali, Tanya Basharat, Mohammed Akhlak Rauf, Aneela Nadeem, Heidi Christensen, Daniel J. Blackburn

**Affiliations:** ^1^ Department of Neuroscience University of Sheffield Sheffield UK; ^2^ School of Computer Science University of Sheffield Sheffield UK; ^3^ Devices for Dignity NIHR MedTech and In Vitro Diagnostics Co‐Operatives Sheffield UK; ^4^ Israac Community Centre Sheffield UK; ^5^ Sheffield Chinese Community Centre Sheffield UK; ^6^ ShipShape Community Centre Sheffield UK; ^7^ Meri Yaadain Bradford UK

**Keywords:** bilinguals, cognitive assessment, dementia, ethnic minority groups

## Abstract

**Background:**

Pen‐and‐paper cognitive assessment tools to detect dementia have higher rates of misdiagnosis amongst minority populations, especially those who complete the assessment in their second language. CognoSpeak is an automated cognitive assessment tool that uses machine learning to detect early signs of cognitive impairment from speech. We assess the utility of different pen‐and‐paper cognitive assessments and CognoSpeak in ethnic minority populations living in the UK.

**Methods:**

Research champions from four community centres across Yorkshire recruited cognitively healthy adults from their community: 51 Somali, 50 South Asian (South Yorkshire), 50 Chinese, and 49 South Asian (West Yorkshire). Participants completed the Montreal Cognitive Assessment (MoCA), Rowland Universal Dementia Assessment Scale (RUDAS), Multicultural Cognitive Examination (MCE), and CognoSpeak.

**Results:**

A high percentage (47.5%) of participants recruited from ethnic minority community centres were misclassified as cognitively impaired with the MoCA, compared to just 3.4% in the RUDAS and 2% in the MCE. An acoustic‐based SVM model analysis of responses to CognoSpeak achieved 83% accuracy in the ethnic minority cohort, at a similar rate to monolinguals (86%). Linguistic and text‐based models showed higher levels of bias.

**Conclusion:**

Cognitive assessments, such as the MCE and RUDAS, may be superior to the MoCA in multilingual ethnic minority populations. Automated AI tools like CognoSpeak show promise in reducing healthcare burden in detecting dementia; however, additional work is required on managing implicit bias in any AI model before they could be clinically implemented.

## Key Points

- The MoCA is less appropriate than the RUDAS and MCE when assessing bilinguals from ethnic minority groups.
- Supermarket fluency is a more linguistically accessible verbal fluency task to test cognition in individuals' second language.
- Automated cognitive assessments show promise as tools to detect early signs of impairment, but need to be developed with bilingual and multilingual communities.

## 1 Introduction

Dementia prevalence is increasing globally [1, 2], with the greatest rise predicted amongst ethnic minority communities, including South Asian and Black populations, suggested to be in part related to the higher prevalence of biological and socioeconomic risk factors within these groups [3]. In the UK, there is a growing ethnic minority population, with nearly 1 in 4 people (25.6%; 15.2 million) identifying as part of a minority ethnic group [4]. Understanding whether cognitive tests to detect dementia and mild cognitive impairment (MCI) are equally accurate across ethnicities is therefore essential.

To diagnose MCI or dementia, clinicians administer standardized cognitive assessments such as the Montreal Cognitive Assessment (MoCA; [5]) or the Addenbrooke's Cognitive Examination (ACE‐III; [6]). Both tests show high sensitivity and specificity in detecting early stages of cognitive impairment [7, 8]. A recent systematic review and meta‐analysis found that the MoCA has a sensitivity of 93.7% and a specificity of 58.8% when screening for MCI in mixed clinical populations [8].

However, applying these assessments to patients from minority ethnic backgrounds and those who speak English as an additional language (i.e., where English is not the individual's first language) can be problematic, often misclassifying healthy individuals in these groups as cognitively impaired [9, 10, 11, 12, 13, 14, 15]. In an American cohort, Ratcliffe et al. [15] found that the MoCA misdiagnosed healthy Black participants as cognitively impaired at twice the rate of White participants. Similarly, in a review of the barriers to Dementia assessments in the UK, Khan and Tadros [10] highlighted that the Mini‐Mental State Exam (MMSE), part of earlier versions of the ACE‐III (ACE‐R), falsely identified 42% of Black individuals as cognitively impaired compared to only 6% of White individuals as determined by a clinical evaluation [11]. This bias highlights the need to develop and use assessment tools effective across diverse ethnicities and language proficiencies. It is important to note that ethnicity in and of itself is not necessarily the determinant of inequalities in dementia assessment accuracy, but rather as a factor related to cultural and linguistic diversity, which contributes towards the inaccuracy.

The Rowland Universal Dementia Assessment Scale (RUDAS; [16]) and its extension, the Multicultural Cognitive Examination (MCE; [17]), may address these limitations, with studies showing lower susceptibility to language and culture‐based biases compared to the ACE‐III and MoCA [17]. Specifically, the RUDAS and MCE do not rely on education and language proficiency‐dependent skills such as language repetition and mathematical procedures (serial subtraction) seen in the MoCA and ACE‐III, resulting in improved reliability of diagnostic screening in linguistically and ethnically diverse populations [18, 19].

For patients who speak English as an additional language, language‐based tasks such as sentence repetition and verbal fluency tasks (VFT) can result in lower scores and potential misdiagnoses. VFTs such as semantic fluency (generating words within a category like animals or items bought at a supermarket) and phonemic fluency (words starting with a specific letter like “F,” “P,” or “S”) are key in detecting and diagnosing MCI and dementia (See Appendix Table 1).

Over 5 million individuals in the UK (8.9%) speak English as an additional language, with more than 1 million (1.8%) reporting little or no proficiency, limiting their accurate assessment and diagnosis in memory clinics. Whilst ideally, patients could complete assessments in their preferred language, translated assessments do not fully resolve this problem, as interpreters facilitating the test may obscure instructions and responses, skewing clinicians' perceptions of the patient [see 20 for review]. Instead, developing and implementing cognitive assessment tools that can be reliably administered by clinicians across diverse demographics and language abilities is vital to adequately support this growing population.

CognoSpeak, an automated language‐based memory assessment tool, was created to investigate whether an at‐home online test could identify individuals requiring referral to memory clinics [21]. Initial research indicates approximately 80%–87% accuracy in distinguishing between individuals with dementia or MCI and healthy controls, although this was based on a predominantly White, monolingual cohort [22, 23, 24]. CognoSpeak uses artificial intelligence (AI) and machine learning algorithms to detect memory issues from speech. Training AI on a largely White, English‐speaking population could introduce bias and fail to represent the diversity of potential users accurately. As a result, we collaborated with four ethnic minority community centres to address these research goals.

1. Assess the misclassification rate of MoCA, RUDAS, MCE in cognitively normal adults from ethnic minority groups across language backgrounds
2. Assess the misclassification rate of CognoSpeak in cognitively normal adults from ethnic minority groups across language backgrounds
3. Compare CognoSpeak's rate of misclassification with that of MoCA, RUDAS, and MCE in this cohort

Given prior evidence of poor MoCA diagnostic accuracy across ethnic minorities [9, 10, 11, 12, 13, 14, 15], and the development of RUDAS and MCE for better cross‐cultural applicability [16, 17], we hypothesise that the MoCA will misclassify individuals in minority groups as cognitively impaired at a higher rate compared to RUDAS and MCE. Based on findings from Pahar et al. [24], we also expect that CognoSpeak will show reduced classification accuracy in minority participants.

## 2 Materials and Methods

### 2.1 Participants

Research Champions from four community centres (Israac Somali Community, Shipshape South Asian Community (South Yorkshire (SY)), Meri Yaadain South Asian Community (West Yorkshire (WY)), and Sheffield Chinese Community Centre) were employed and trained to use the MOCA, RUDAS, MCE, and CognoSpeak. Participants had to be judged by the research champion as sufficiently proficient in English to complete the cognitive assessments in English. We initially targeted participants over the age of 50, but due to the limited English proficiency (and availability) of older members of the community, this was widened to anyone over 18. Hence inclusion criteria was broadened to cognitively intact adults from an ethnic minority community, who were proficient enough in English to complete all assessments in English. Participants received a £25 gift card as compensation for their time.

205 participants were recruited; however, 5 participants failed to complete one or more of the cognitive assessments, meaning 200 were included (see Table 1).

**TABLE 1:** Participant demographics.

Community centre	*n*	Age	Education	Age learning English	Gender		English fluency					Ethnicity	First language (excluding English)
		*M (*SD)	*M (*SD)	*M (*SD)	Male	Female	Basic	Good	Very good	Fluent	First language		
Somali	50	41.6 (14.1)	13.7 (6.0)	11.3 (9.2)	23	27	2	6	18	18	6	47 Black African 3 mixed: White and Black African	43 Somali 1 French
Chinese	50	55.8 (10.1)	16.0 (3.7)*	7.7 (3.4)	18	32	3	23	12	5	7	50 Chinese	17 Chinese 14 Cantonese 11 Mandarin
South Asian (SY)	51	50.8 (8.8)	12.2 (4.3)	4.9 (0.8)	14	37	3	14	1	15	18*	48 Pakistani 1 mixed: White and Asian 1 Other Asian	26 Urdu 7 Punjabi 1 Pashto 1 Arabic
South Asian (WY)	49	54.3 (11.7)	14.3 (3.4)	6.0 (3.4)	16	33	0	20	3	0	26	41 Pakistani 7 Indian 1 Other Asian	16 Urdu 1 Hindi 1 Mirpuri 5 Punjabi
Total	200	50.3 (12.7)	13.9 (4.7)	7.4 (5.7)	71	129	8	63	34	38	57	—	—

*Note:* Education was not recorded for 8 participants from the Chinese community. Ethnicity was not reported for 6 participants from South Asian (WY) (West Yorkshire) community. Two Participants from the South Asian (SY) (South Yorkshire) community centre recorded themselves as native English speakers with different first language.

### 2.2 CognoSpeak Assessment

Participants completed the online automated cognitive assessment: CognoSpeak; consisting of 14 questions prompting verbal responses, which were audio‐recorded and manually transcribed. Anxiety and mood were assessed using the GAD7 [25], and PHQ9 [26].

#### 2.2.1 Procedure

Participants completed: RUDAS, MCE, and MoCA with research champions. An online link was sent to the participant to complete CognoSpeak using their laptop, computer, or iPad. CognoSpeak was completed with the aid of the community centre research champions in the case of technical difficulties or preference (see Appendix Table 2). Research champions or the participant themselves rated participants' conversational English language proficiency on a five‐point scale from Basic (1), Good (2), Very Good (3), Fluent (4), and First Language (5), and collected demographic information. All assessments were completed in English only.

#### 2.2.2 Analysis

To analyse the appropriateness of the different pen and paper cognitive assessment tools, we created a generalised logistic mixed effects regression model including a random intercept for the individual. In each model, the binary dependent variable was whether the participant scored above or below the assessment threshold defined by the assessment administration guidelines to be classed as cognitively normal (MCE: ≥ 70/100 [17], MoCA ≥ 26/30 [5], RUDAS ≥ 23/30 [16]). For the MoCA, an additional analysis using the less conservative MoCA threshold of ≥ 23/30 suggested by Carson et al. [27] was conducted. Estimates of the significance of fixed effects were derived from likelihood ratio tests, comparing models with and without those effects.

A linear mixed effects model was used to determine the effect of different demographic and mood factors on individual's scores in each pen and paper assessments.

To determine the effect of language proficiency on verbal fluency performance across the pen and paper assessments, a verbal fluency score was calculated based on the number of words produced within each VFT minus the number of errors made (repetitions, proper nouns, and intrusions).

#### 2.2.3 CognoSpeak

To assess the accuracy of CognoSpeak for individuals who speak English as an additional language, we developed a fully automated pipeline to identify cognitive decline from audio recordings. First, transcripts were generated from participants' audio recordings using an automatic speech recognition system (Whisper [28]). Then, using a large language model (LLM)‐based model, we fine‐tuned it to a downstream task as a sequence classifier to detect cognitive decline. We extracted both acoustics and linguistic features to train a traditional classifier such as a support vector machine (SVM), and fine‐tuned the LLMs. Both classifiers produced two diagnostic labels: cases (dementia and MCI) and controls [24]. The trained architectures were tested on the 200 bilingual participants (Table 1) and 219 monolingual healthy participants (see Table 2 of [24]).

## 3 Results

**FIGURE 1:**
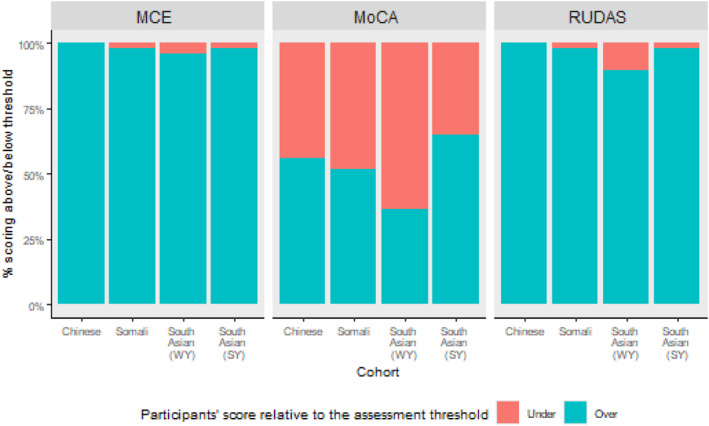
Percentage of Participants who Reached each Assessment Tool's “Cognitively Normal” Threshold.

Despite all ethnic minority participants being cognitively intact, only 52.5% reached the MoCA threshold for cognitive normality (Figure 1). This was significantly lower (*χ* ^2^(2) = 15.67, *p* < 0.001) than when the same participants completed RUDAS (96.5%; *Z* = 3.95, *p* < 0.001), and MCE (98.0%; *Z* = 3.65, *p* < 0.001). Across assessments, pass rate significantly decreased with age (*χ* ^2^(1) = 9.93, *p* = 0.002), fewer years of education (*χ* ^2^(1) = 10.12, *p* = 0.001), and lower English language proficiency (*χ* ^2^(4) = 11.74, *p* = 0.019). There was no significant relationship between anxiety score (*p* = 0.92) or depression score (*p* = 0.41), on passing assessment thresholds.

**FIGURE 2:**
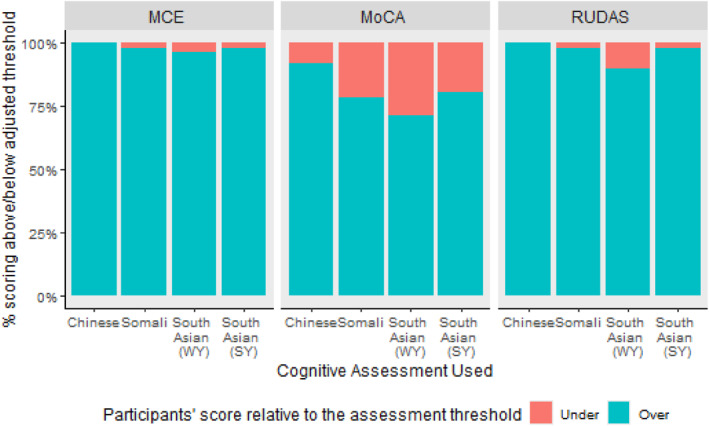
Percentage of Participants who Reached each the Adjusted “Cognitively Normal” Threshold.

When applying a less conservative MoCA cutoff of ≥ 23 [27], correct identification of cognitive normality rose from 52.5% to 80.5% (Figure 2). This was still significantly lower (*χ* ^2^(2) = 68.47, *p* < 0.001) than when the same participants completed RUDAS (*Z* = 7.87, *p* < 0.001), and MCE (*Z* = 6.84, *p* < 0.001). However, English proficiency was no longer related to meeting the assessment threshold (*χ* ^2^(4) = 7.04, *p* = 0.13), indicating increased accessibility. Both age and years in education remained significant predictors of passing the assessment threshold, however this relationship was less significant for age (*χ* ^2^(1) = 5.8, *p* = 0.016), but more significant for years of education (*χ* ^2^(1) = 15.01, *p* < 0.001).

**FIGURE 3:**
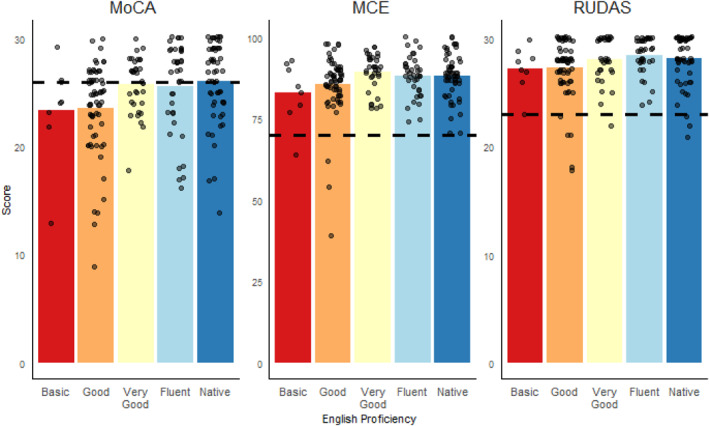
Participants' Cognitive Assessment score relative to their English Proficiency. Note. Dotted line indicates Assessment Threshold for Cognitive Impairment.

A linear regression model revealed that across all assessments, participants' scores significantly decreased with age (MoCA: *β* = −0.05, *p* = 0.03; MCE: *β* = −0.17, *p* < 0.001, RUDAS *β* = −0.04, *p* = 0.001), and increased with estimated years of education (MoCA: *β* = 0.23, *p* < 0.001; MCE: *β* = 0.48, *p* < 0.001; RUDAS: *β* = 0.10, *p* = 0.002). As shown in Figure 3, higher English proficiency was a significant positive predictor of scores on the MoCA (*β* = 0.68, *p* = 0.001), but not the MCE (*β* = 0.61, *p* = 0.13), nor RUDAS (*β* = 0.22, *p* = 0.07).

These results support our hypothesis, indicating that the MoCA is less appropriate for patients from ethnic minorities, especially those less proficient in English.

### 3.1 Verbal Fluency Tasks

As sphericity was violated (*p* < 0.001), a repeated measures two‐way ANOVA with a Greenhouse‐Geisser correction was used to investigate the relationship between English proficiency, verbal fluency tasks, and their interaction on participants' fluency score. Simple main effects analysis showed that mean score was significantly different across English proficiency levels (F(4, 191) = 10.15, *p* < 0.001), and across VFTs (F(4, 764) = 94.61, *p* < 0.001). Moreover, there was a significant interaction between English proficiency and the fluency task on participants' performance (F(16,764) = 3.64, *p* < 0.001).

**FIGURE 4:**
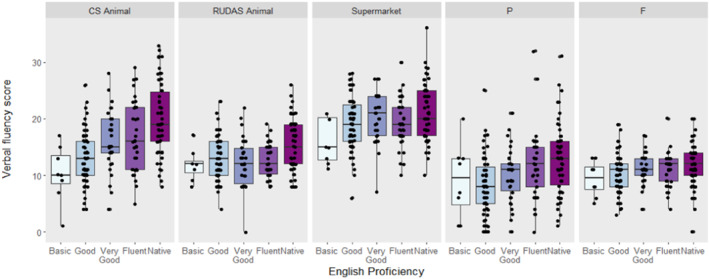
Bilingual participants' mean fluency test scores relative to English proficiency.

A post hoc Spearman's correlational analysis revealed that participants' English proficiency had the highest correlation with their performance on CognoSpeak's Animal fluency (*r* = 0.45, *p* < 0.001). Only the supermarket fluency task did not demonstrate a significant correlation between participants' English proficiency and their score (*r* = 0.14, *p =* 0.051) (Figure 4). Taken together, VFTs are differentially accessible to multilingual individuals, with performance on the supermarket fluency task the least impacted by English proficiency.

Unlike in the pen and paper assessments, a multiple linear regression predicting verbal fluency task performance found that higher depression scores (PHQ‐9) were associated with significantly lower average VFT scores (*β* = −0.22, *p* = 0.008), whereas higher anxiety scores (GAD‐7) were associated with significantly higher mean VFT scores across tasks (*β* = 0.19, *p* = 0.033).

### 3.2 CognoSpeak Assessment

As illustrated in Table 2, the SVM model combined with acoustic features produces the most consistently accurate labels across cohorts, which accurately identified 84% participants as healthy. This performed worse with South Asian bilinguals with a West Yorkshire accent. In contrast, the SVM classifier with linguistic features performed poorly for all bilingual cohorts despite a similar accuracy for English monolinguals. A similar trend is seen in the LLMs, which are directly fine‐tuned on the automatically Whisper‐generated transcript. Overall, the latter two architectures performed much poorer, with an accuracy of 59%.

**TABLE 2:** Accuracy of 2‐way classification models (Healthy vs. Cognitively Impaired) using responses to all questions in CognoSpeak.

	Acoustics + SVM	Linguistics + SVM	Text + LLM
Monolinguals	86%	86%	88%
Somali	84%	45%	51%
Chinese	90%	58%	56%
South Asian (WY)	64%	56%	51%
South Asian (SY)	95%	51%	50%
Overall	84%	59%	59%

*Note:* Table shows the percentage of participants from each cohort correctly classified as healthy in each binary classification model, with green showing higher classification accuracy, and red showing lower classification accuracy. LLM‐ large language model. SVM‐ Support Vector Machine.

## 4 Discussion

Our study examined the accuracy of various pen‐and‐paper cognitive assessments and an online automated assessment (CognoSpeak) amongst a cognitively intact bilingual ethnic minority population living in the UK. The MoCA misclassified nearly half of cognitively healthy participants from ethnic minority groups as cognitively impaired. In contrast, the MCE and RUDAS misclassified fewer than 4% of participants as cognitively impaired, suggesting these assessments may be more suitable for culturally and linguistically diverse groups. This seems true regardless of the participants' subjective English proficiency, with only the MoCA displaying a significant correlation between English skills and scores. CognoSpeak demonstrates the ability to maintain high classification accuracy for these cohorts, though caution needs to be taken when developing the AI architecture to identify this.

Our findings align with previous research, indicating that the MoCA is less suitable for people from an ethnic minority taking the assessment in their second language. Previous research has suggested that adopting a lower MoCA cutoff score of ≥ 23, rather than the suggested ≥ 26, may help to combat the high incidence of false positives especially amongst ethnic minority populations and individuals with fewer years of education [14, 27]. Whilst adjusting the threshold for underrepresented groups can reduce false positives, as we see in our research also, it does not address the fundamental issue of using the MoCA in multicultural contexts. Many cognitive assessments are based on normative data from white individuals taking the test in their native language, without accounting for additional cognitive demands faced by those completing it in a different language. We found that even when lowering the threshold, almost one in 5 participants were misclassified as cognitively impaired. Hence, clinicians should consider using alternative assessments developed with multicultural and linguistically diverse populations in mind, such as the MCE or RUDAS, especially when assessing patients who do not speak the dominant language of the country as their first language.

As an alternative to adapting current manual methods of assessment, our results demonstrate the potential for automated assessments for individuals across language backgrounds and cultures. When using an acoustics‐based analysis of participants' speech, CognoSpeak was able to correctly identify healthy participants regardless of their language background with a high rate of accuracy. However, text and language based automated analysis of participants' responses was significantly worse at recognising the cognitive status of bilinguals compared to monolinguals, highlighting the potential bias that may creep into these systems if not developed with cultural and linguistic diversity in mind. This concern is common across many speech technologies [29], and has been highlighted as a limiting factor to the adoption of this technology adoption amongst General Practitioners [30]. Nonetheless, even the poorest performing architecture shows a similar classification accuracy to the MoCA in these cohorts, indicating that whilst this technology needs to continue developing with more diverse cohorts before it can be clinically implemented, the current tools we rely on are equally if not even less appropriate.

The choice of VFT need to consider the influence of a patient's first language on their performance. Some Somali participants mentioned specific language barriers affecting their performance. For example: *“A letter of P. … I've got a language barrier, I think. Um … we don't have that, you know P, you know, in our language*.” In Somali and Arabic, the phoneme/p/does not exist [31], so responses to ‘P’ word fluency tasks are sometimes unintentionally replaced with ‘B’ words in English [32]. This was observed amongst our participants, many of whom responded with “B” words not due to memory or language fluency issues, but because of phonological barriers. For example, one participant responded with all but one “B” word to the “P” word task “*Book, bravo, bank, bye, balloon, pants*”. Waheed et al. [33] also highlighted this issue that has been raised when translating the ACE‐III into Egyptian‐Arabic, wherein “P” words needed to be replaced with “Sh” sounds.

Amongst the VFTs used, supermarket fluency proved most accessible for bilingual participants at all levels of English proficiency. The better results may be due to fewer language barriers or different cognitive strategies. Learning supermarket items in English is essential for daily independence, unlike animals, which are less relevant to everyday communication. Frequent exposure to words facilitates second language acquisition [34], so bilinguals might face fewer linguistic challenges when performing fluency tasks with familiar categories encountered out of necessity. Hence, this may provide a more accurate reflection of their verbal fluency and cognitive abilities.

## 5 Limitations and Future Directions

Whilst this research highlights the bias in current cognitive assessments, there are some key limitations to our findings. Firstly, the average age of our cohort is lower than that of most patients seeking a memory diagnosis [35]. However, the insight that even young healthy participants struggled to meet the MoCA threshold highlights issues in its use in populations who speak English as an additional language. Secondly, we only investigated the appropriateness of these assessments in cognitively normal individuals, leaving it unclear the likelihood of false negatives in these cohorts. Whilst our study highlights the high risk of false positives when using these assessments, it is unclear whether these more accessible assessments are actually missing potential early signs of cognitive impairment, limiting their utility. Hence, future research should build upon these findings, looking at the appropriateness of different cognitive assessment tools for patients with cognitive impairment across language backgrounds and ethnicities. This would enable us to see the clinical utility of both culturally accessible, and automated tools in the detection of dementia.

## 6 Conclusion

The MoCA appears less appropriate than the MCE and RUDAS in assessing cognition in bilingual ethnic minority individuals. Automated cognitive assessment tools, like CognoSpeak, show promise in detecting the cognitive status of individuals across language backgrounds, but need to be codeveloped and incorporate normative data from minority groups and bilinguals and multilinguals to reduce the risk of bias. Future work needs to address the risk of false negative across these assessment tools, investigating the accuracy of these tools in patients with MCI and Dementia across underrepresented groups.

## Funding

This work was supported by the NIHR i4i grant [NIHR202911, 2022].

## Ethics Statement

This research was approved by the NHS Health Research Authority (IRAS:277,394) on 01/12/2022.

## Conflicts of Interest

The authors declare no conflicts of interest.

## Data Availability

The data that support the findings of this study are available from the corresponding author upon reasonable request.

## Appendix

**TABLE A1:** The type of verbal fluency assessment used in each of the Cognitive assessments and its associated score.

Cognitive assessment	Verbal fluency tasks	Verbal fluency task points		Total score	Cut‐off score
ACE‐III	Semantic (Animal) + phonemic (P)	14	14%	100	≥ 88 or 82
MoCA	Phonemic (F, S, or B)	1	3.3%	30	≥ 26
RUDAS	Semantic (Animal)	8	26.7%	30	≥ 23
MCE	Semantic (supermarket) + RUDAS semantic (Animal)	36	36%	100	≥ 70

**TABLE A2:** The location of the CognoSpeak assessments and whether help was required to complete the assessment across community centres.

		Location of CognoSpeak Assessment				Was help required to complete CognoSpeak?		
Community centre	*n*	Community centre	Home	Undisclosed	Other	With help	By Self	Undisclosed
		*n* (%)	*n* (%)	*n* (%)	*n* (%)	*n* (%)	*n* (%)	*n* (%)
**Somali**	50	26 (52%)	13 (26%)	7 (14%)	4 (8%)	5 (10%)	36 (72%)	9 (18%)
**Chinese**	50	16 (32%)	17 (34%)	17 (34%)	0 (0%)	9 (18%)	40 (80%)	1 (2%)
**South Asian (SY)**	51	34 (66.7%)	9 (17.6%)	8 (15.7%)	0 (0%)	14 (27.5%)	37 (72.5%)	0 (0%)
**South Asian (WY)**	49	49 (100%)	0 (0%)	0 (0%)	0 (0%)	32 (65.3%)	14 (28.6%)	6 (6.1%)
**Total**	200	125 (62.5%)	39 (19.5%)	32 (16%)	4 (2%)	60 (30%)	127 (63.5%)	13 (6.5%)

*Note:* The location “Other” refers to: workplace (*n* = 2), publick space (*n* = 1), and Mosque (*n* = 1).
